# Predictive Chemical Kinetic Modeling: Where We Succeed, Where We Struggle, and What Comes Next

**DOI:** 10.1021/acsengineeringau.5c00091

**Published:** 2026-01-06

**Authors:** Alon Grinberg Dana, Kevin M. Van Geem, Carlo Cavallotti, William H. Green

**Affiliations:** † Wolfson Department of Chemical Engineering, 26747 Technion − Israel Institute of Technology, Haifa 3200003, Israel; ‡ Grand Technion Energy Program (GTEP), 26747 Technion − Israel Institute of Technology, Haifa 3200003, Israel; ¶ Boeing-Technion SAF Innovation Center, 26747 Technion − Israel Institute of Technology, Haifa 3200003, Israel; § Laboratory for Chemical Technology, Department of Materials, Textiles and Chemical Engineering, Faculty of Engineering and Architecture, Ghent University, Technologiepark 125, B-9052 Zwijnaarde, Belgium; ∥ Department of Chemistry, Materials, and Chemical Engineering “G. Natta”, 18981 Politecnico di Milano, Milano 20133, Italy; ⊥ Department of Chemical Engineering, 2167 Massachusetts Institute of Technology, Cambridge, Massachusetts 02139, United States

**Keywords:** predictive chemical kinetics, automated mechanism generation, machine learning, heterogeneous catalysis, electrochemistry, ab initio calculations

## Abstract

Chemical kinetic modeling plays a foundational role in fields ranging from energy to environmental science, pharmaceuticals, and advanced materials. The past two decades have seen remarkable progress, particularly in modeling gas-phase reactions for thermochemical processes, leading to impactful industrial applications such as steam cracking and air quality management. However, new challenges are emerging. The successful development of systematic methodologies for the description of gas-phase kinetics opens the possibility to apply the same approach to the study of more challenging systems. Here, we review recent advances, including ab initio transition state theory-based master equation estimation of elementary rates, automated mechanism generation, machine-learning-assisted kinetics, and uncertainty quantification, and discuss the advances needed to apply the same methodological approach in areas such as heterogeneous catalysis, electrochemistry, liquid-phase and solid-state reactivity, and multiscale model integration. We advocate for the development of targeted tools, especially methods that go beyond empirical tuning toward first-principles-based predictions. We highlight the need for accessible software and AI-augmented workflows to democratize modeling for industry and academia alike. In this perspective, we call attention to not only what has worked but also what remains unsolved, advocating to avoid overemphasizing successes in scientific works at the expense of realism. The next decade should focus on predictive capability, physical accuracy, and community infrastructure (e.g., databases and services) to enable innovation across diverse fields. We argue that kinetic modeling, properly equipped, can accelerate discovery far beyond its traditional domains.

## Graphical abstract

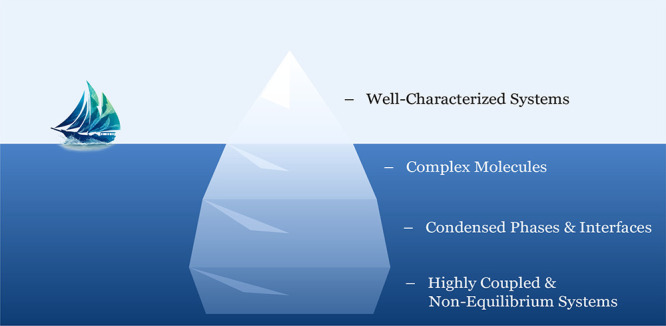

## 1 Introduction

Chemical kinetic modeling plays a central role in the design, optimization, and interpretation of chemical processes,^1,2^ spanning combustion,^3,4^ catalysis,^4–7^ environmental protection,^8^ polymer processing and degradation,^9^ pharmaceuticals,^10,11^ and chemical synthesis. In recent decades, kinetic models have contributed to major scientific and societal advances: improving urban air quality, guiding the development of cleaner combustion technologies,^12^ and enabling more efficient industrial processes such as steam cracking, refining processes, and methane reforming. These achievements are rooted in the deep integration of physical chemistry, reaction engineering, and computational modeling, supported by a robust ecosystem of theory, algorithms, and software.

The successes of predictive chemical kinetic modeling have been enabled by a systematic methodological framework that links ab initio theory, statistical mechanics, rate theory, databases, and validation, as summarized in Figure 1. Molecular properties derived from quantum chemistry underpin reliable thermochemistry and rate coefficients through statistical mechanics and Transition State Theory (TST)/Master Equation (ME) calculations.^13,14^ These in turn feed into curated databases and core mechanisms, which are assembled – often automatically – into detailed chemical kinetic models. Data consistency is preserved using multiscale informatics.^15^ Crucially, iterative validation against species-resolved and reactor-scale experiments ensures that the models remain quantitatively anchored to reality. This cycle of theoretical computations and experimental validations has provided the rigor and reproducibility that make predictive modeling possible, and it forms the foundation on which progress in new domains must build.

**Figure 1:**
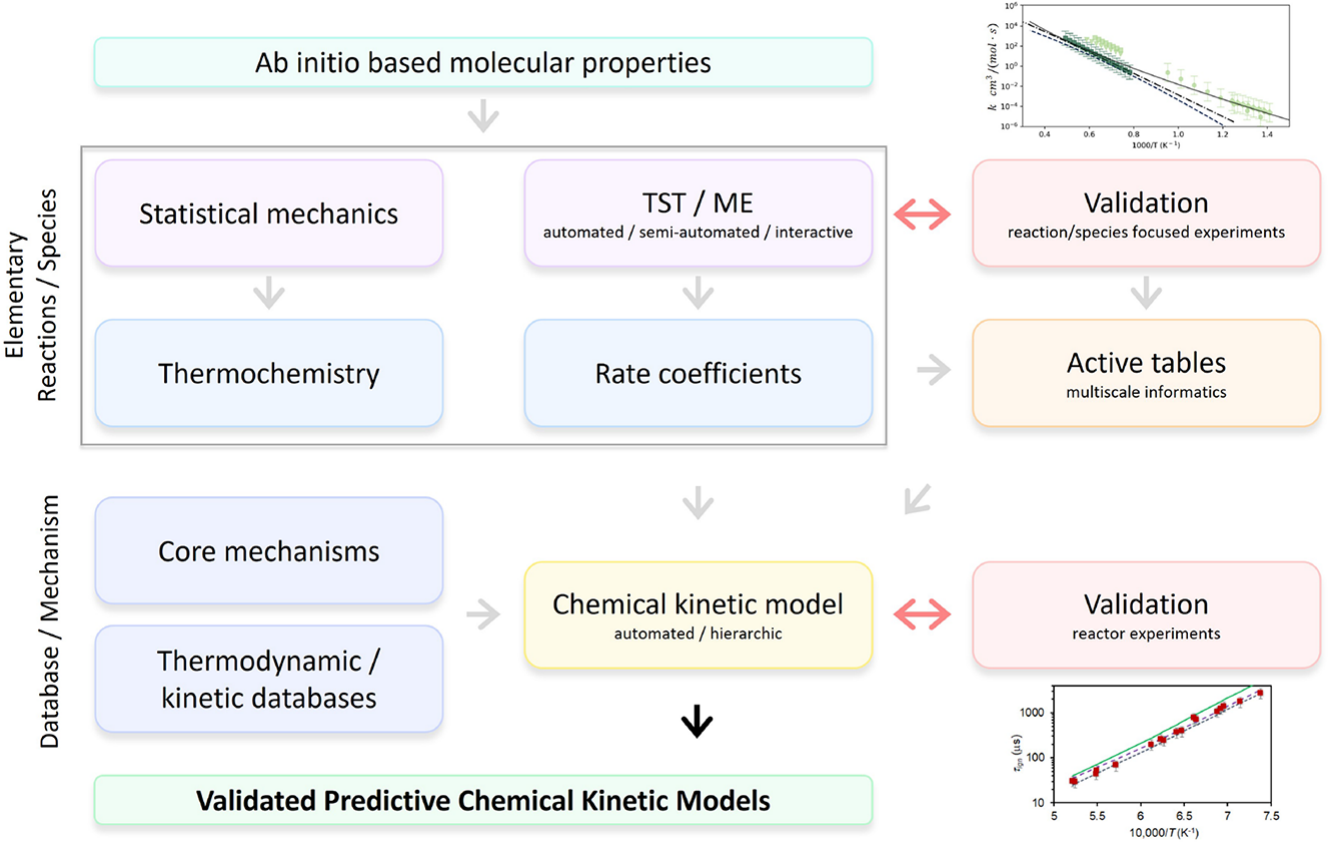
Workflow for constructing validated predictive chemical kinetic models. Ab initio molecular properties, usually determined through density functional theory (DFT) or wave function theory calculations, feed into statistical mechanics and TST/ME calculations to generate thermochemistry and rate coefficients. Chemical kinetic models are assembled either automatically or in a hierarchic approach based on core mechanisms and available thermo-kinetic data. Iterative validation occurs both at the level of individual reactions/species and at the reactor scale for speciation and global observables. The process yields predictive and experimentally benchmarked chemical kinetic models.

The demands on kinetic modeling are now shifting toward systems of unprecedented complexity, spanning multiple phases, time scales, and length scales. Applications extend far beyond traditional gas-phase kinetics into electrochemical,^16^ plasma,^17^ slurry-phase, and heterogeneous catalytic systems,^5^ as well as emerging domains such as plastic recycling,^18,19^ cancer metabolism and drug synthesis,^20–23^ and pharmaceutical reaction engineering.^10,11^ Simultaneously, experimental capabilities have advanced dramatically: we can observe more species with greater precision, resolve more transient intermediates, and generate richer data sets, reducing measurement uncertainty and raising expectations for model accuracy, scalability, and scope.

These trends present both an opportunity and a challenge. While most kinetic models have been developed and validated within relatively narrow operating domains, the present moment demands approaches that are more general, quantitatively predictive across broader chemical space,^2^ and more automated through robust integration of theory, data, and computation.^24–26^ Yet many limitations in current practice remain underexamined. As a community, we often emphasize successes while minimizing attention to failures, systematic errors, or fundamental gaps in our tools.

In parallel, advances in machine-learning (ML)^27^ are reshaping the predictive landscape, enabling exploration of vast chemical reaction spaces,^28^ improving estimates of thermochemical and kinetic parameters,^29–32^ accelerating mechanism generation, and enabling direct inference of reaction networks from experimental data, e.g., in time-resolved spectroscopy^33^ and in large-scale intracellular metabolic systems integrating multiomics data sets.^34^ These developments illustrate a broader shift toward automation, machine-assisted modeling, and confidence-aware predictions. However, significant blind spots remain: heterogeneous catalysis on realistic morphologies,^35^ liquid-phase chemistry with explicit solvation, electrochemical systems with complex interfaces, plasma-assisted processes, and surface chemistry involving diverse adsorbate–active site interactions^36^ are still underrepresented.

In this Perspective, we critically assess the current state of chemical kinetic modeling and discuss the challenges that must be met to extend the methodological approach successfully developed and applied in the study of processes controlled by gas phase kinetics, such as combustion, to other fields. We highlight both the successes and the frontiers where progress is needed, in theoretical methods, data integration, and software infrastructure. We advocate for a modeling culture that is open, realistic, and predictive, fostering automated workflows, rigorous uncertainty quantification (UQ), and democratized tools, meaning sharing open-source software, publishing replay scripts along with mechanisms where relevant, and noting the version of the used tools. As the community confronts urgent global challenges in sustainable energy, climate change, materials discovery, and environmental stewardship, chemical kinetics must evolve to deliver insight not just retrospectively, but proactively, across an expanding range of increasingly complex systems.

## 2 What We Can Do Well

Kinetic modeling has achieved notable success in accurately predicting the behavior of relatively complex reacting systems across multiple fields. Below we highlight examples from combustion, catalysis, atmospheric chemistry, electrochemistry, and biomass conversion (Figure 2) where detailed kinetic models have demonstrated strong predictive capabilities, backed by experimental validation. These successes generally arise in settings that are idealized or well-characterized, where mechanisms, thermochemistry, transport, and material structure are sufficiently constrained to support quantitative prediction. At the same time, many of these domains straddle the boundary between “success” and “challenge”: as model assumptions are relaxed and we move from well-defined testbeds to realistic, multicomponent, multiphase, and multiscale operating environments, predictive accuracy typically degrades (see Section 3).

**Figure 2:**
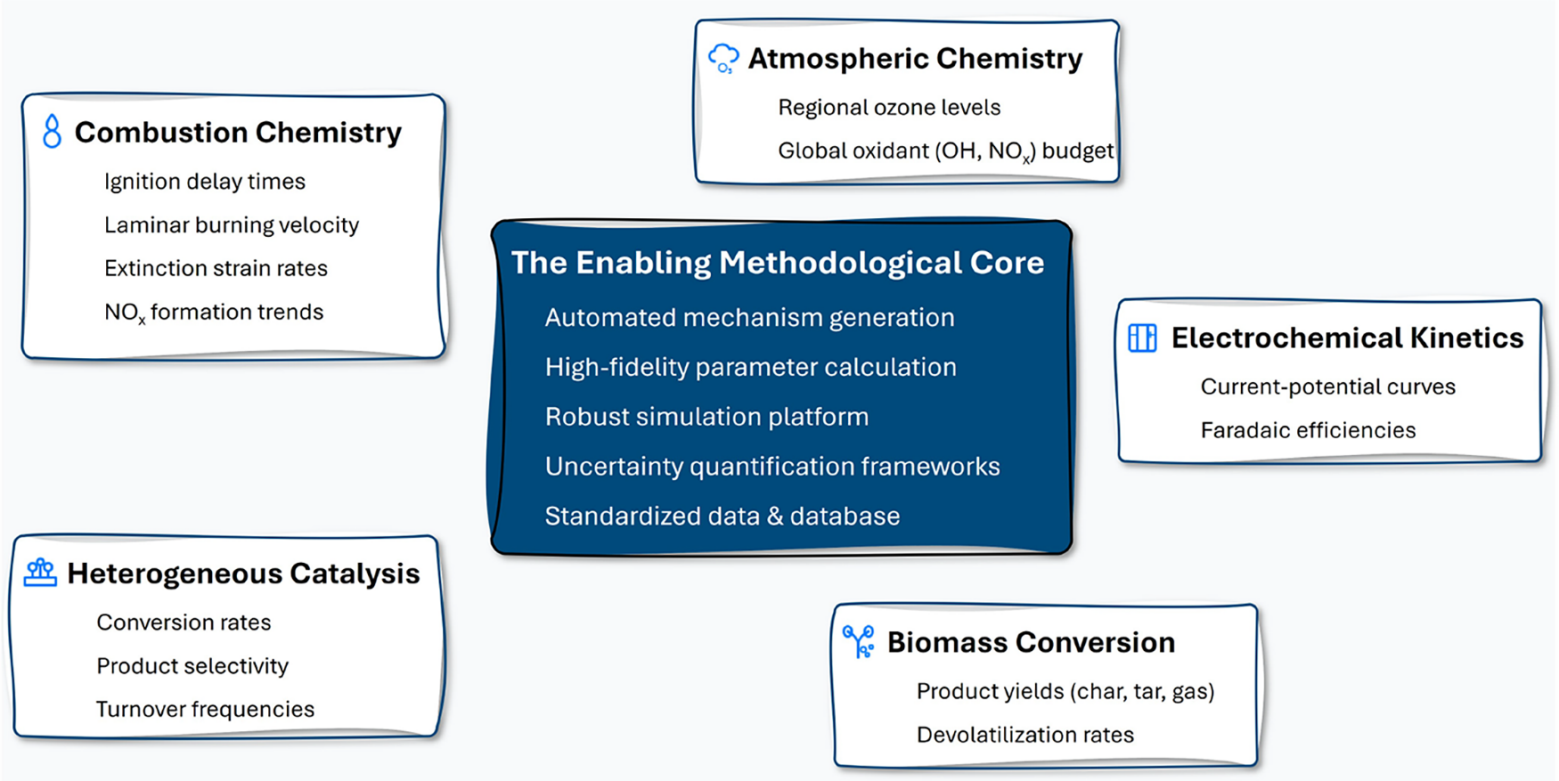
The enabling methodological core of modern chemical kinetics and its successful application across diverse predictive domains.

### 2.1 Combustion Chemistry

Combustion chemistry is the field in which the methodological approach schematized in Figure 1 was initially developed, continuously refined, and reached maturity, finally evolving into a quantitative predictive tool. In combustion science, detailed chemical kinetic mechanisms can reliably predict fundamental combustion properties for small fuels, as illustrated by the representative validation results in Figure 3. For example, the Foundational Fuel Chemistry Model (FFCM v. 1 and 2)^37^ for methane reproduces laminar flame speeds in methane–air mixtures across wide conditions, in close agreement with measurements.^38^ At engine-relevant temperatures and pressures, FFCM-1’s flame propagation rates align with most literature data sets, and ignition delay times of simple fuels (e.g., H_2_, CH_4_, and their mixtures) computed from state-of-the-art mechanisms often fall within experimental uncertainties.^39,40^ The state of the art for the development of chemical kinetic models for thermochemical applications has been recently reviewed by Mehl et al.,^41^ where comprehensive citations to the pertinent literature can be found.

For larger, real multicomponent fuels, the hybrid chemistry (HyChem) approach has emerged as a powerful framework.^43–46^ In HyChem, the pyrolysis or oxidative pyrolysis of the parent fuel is represented by a small number of experimentally constrained lumped reaction steps, while a detailed foundational fuel chemistry mechanism describes the oxidation of the resulting small-molecule fragments. This decoupling enables accurate predictions of ignition delay times, laminar flame speeds, and extinction strain rates for diverse jet fuels, rocket fuels, and gasoline blends.^44,46^ The approach has also been extended to capture negative-temperature-coefficient (NTC) behavior via formaldehyde speciation markers,^46^ and, when coupled with validated NO_*x*_ submodels, reproduces NO_*x*_ formation trends in premixed flames of large hydrocarbon fuels over lean-to-rich conditions.^45^ HyChem mechanisms can be systematically reduced to ∼30 to 40 species for tractable turbulent combustion simulations while retaining predictive fidelity.^44,46^

Automated mechanism generation has become routine in this domain: The Reaction Mechanism Generator (RMG) software constructs large and extensive mechanisms using a rate-based approach with integrated thermochemistry and rate estimation, uncertainty analysis, and pressure-dependence workflows.^4,47–49^ Automated rule-based frameworks such as Genesys^7,50^ further expand coverage to heavier fuels and surfaces. Pressure-dependent kinetics is reliably computed using RRKM/master-equation pipelines (e.g., using Arkane,^51^ MESMER,^52^ MESS,^53^ or Multiwell^54^) with proven falloff treatments.

Despite excellent progress, there are still some aspects of non-Boltzmann kinetics that are not well-represented in most current kinetic models, as discussed at the 2022 Faraday Discussion.^55^

### 2.2 Heterogeneous Catalysis

For prototypical catalytic chemistries on well-characterized surfaces, first-principles microkinetic models routinely reproduce measured selectivities and concentration trends of major observables when key surface physics-coverage effects, e.g., lateral interactions and site balances, are included.^5,6,56^ Mean-field microkinetic models and lattice-based kinetic Monte Carlo (kMC) generally predict consistent trends in the absence of strong lateral interactions. Still, kMC can more accurately capture local coverage effects when such interactions are significant, at the cost of higher computational expense.^57,58^ Descriptor-based screening studies confirm that for many design tasks microkinetic models offer a good balance between accuracy and efficiency, being orders of magnitude faster than kMC while retaining predictive trends.^58^

Goldsmith and West pioneered automated generation of heterogeneous microkinetic mechanisms through the open source RMG-Cat framework,^59^ later merged into RMG.^4^ In its original demonstration on dry reforming of methane (DRM) over Ni, RMG-Cat recovered the dominant network found in an expert-compiled mechanism and proposed additional plausible elementary steps, establishing that automated generation can reproduce and extend curated heterogeneous mechanisms.^59^ As a recent application example, Ritov and Grinberg Dana used a fully automated RMG-based workflow to model Pt-catalyzed DRM; the microkinetic model quantitatively reproduced CH_4_/CO_2_ conversions and syngas production over 700–1100 K and varied feed ratios, and the sensitivity analysis identified OCX (adsorbed CO) as a bottleneck intermediate governing temperature-dependent kinetic regimes.^60^

Selective acetylene hydrogenation on Pd, central to ethylene purification, illustrates the level of mechanistic detail now achievable. Microkinetic analyses that incorporate adsorbate competition and subsurface-H equilibria, together with improved adsorption energetics, have reproduced the observed activity–selectivity trade-offs and composition trends across operating conditions, achieving close theory-experiment parity.^61^

Beyond single systems, the automation ecosystem has broadened. Genesys-Cat automatically generates gas–surface mechanisms and uses Bayesian optimization to estimate missing kinetic parameters from limited data, yielding accurate and thermodynamically consistent models, and demonstrated on processes such as iso-octane cracking on zeolites.^7^ Recent perspectives emphasize hybrid parameter evaluation (first-principles, semiempirical, and data-driven), tighter links to operando spectroscopies, and multiscale coupling to bridge atomistic kinetics with reactor performance.^62–66^

Standardization efforts in surface thermochemistry are accelerating reproducibility and cross-platform use of heterogeneous mechanisms. Kreitz et al.^35^ introduced a unified terminology and algebraic framework for referencing and aligning adsorbate thermochemistry across formats and sources, reducing inconsistencies and enabling seamless reuse within automated microkinetic workflows. Overall, these developments demonstrate that for well-characterized catalytic systems, when combined with robust thermochemistry, appropriate treatment of coverage effects, and validated elementary steps, microkinetic models can quantitatively predict reaction rates, selectivities, and mechanistic regimes, providing actionable insights for catalyst screening and design.

### 2.3 Atmospheric Chemistry

Near-explicit gas-phase mechanisms have been successfully embedded in 3D air quality models to predict regional ozone and secondary pollutant episodes. The Master Chemical Mechanism (MCM) has been integrated at scale and shown to reproduce observed ozone concentrations and diurnal patterns in field campaigns and regional case studies, with performance comparable to or better than condensed regulatory mechanisms when emissions and meteorology are well constrained.^67,68^ These applications demonstrate that thousands of volatile organic compound (VOC) oxidation reactions and intermediates can be carried in Eulerian models with sufficient fidelity to inform policy and source apportionment analyses.^67,68^

Mechanistic improvement in global chemistry-transport models increasingly relies on targeted laboratory and field constraints. For example, recent updates to tropospheric halogen chemistry in the GEOS-Chem model incorporated new HOBr heterogeneous reaction kinetics and mechanistic sea-salt aerosol debromination, resolving long-standing biases in modeled BrO and altering global oxidant budgets.^69^ Bates et al.^70^ implemented the latest JPL/IUPAC kinetic data for nitrogen, hydrocarbon, and halogen reactions, demonstrating that changes in rate constants and branching ratios alone can shift global burdens of O_3_, NO_*x*_, OH, CO, and peroxyacetyl nitrate by several percent. Shah et al.^71^ used aircraft NO/NO_2_/O_3_ observations to evaluate free-tropospheric NO_*x*_ in four global models, identifying missing organic NO_*x*_ pathways and an unrepresented source from particulate nitrate photolysis on sea-salt aerosol. Incorporating this heterogeneous source in GEOS-Chem increased modeled free-tropospheric O_3_ by up to 5 ppbv and OH by nearly 20%, improving agreement with ozonesondes. Process-level kinetic parametrization at the atmosphere-ocean interface can likewise correct large-scale model biases: Pound et al.^72^ replaced a fixed oceanic ozone deposition velocity with a temperature- and iodide-dependent kinetic scheme, halving the global oceanic O_3_ sink, improving marine boundary layer O_3_ predictions, and highlighting the need for mechanistic surface–ocean coupling in global models.

Advances in organic oxidation chemistry are also reshaping atmospheric mechanisms. Barber and Kroll^73^ reviewed how the presence of functional groups in later-generation oxidation intermediates can substantially alter both the energetics and kinetics of key radical reactions (RO_2_, RO, Criegee), opening entirely new pathways that are absent from current mechanism parametrizations. To address this gap, Barber et al.^74^ adapted RMG^4^ to systematically explore OH-initiated oxidation of 200 mono- and bifunctional *n*-pentanes at atmospheric conditions. This automated network generation identified both canonical and uncanonical radical chemistry, atmospherically competitive pathways, such as radical–carbonyl ring-closure to cyclic alkoxy radicals—reaction types not currently represented in most near-explicit models. In parallel, Barber et al.^75^ provided direct laboratory kinetics for unimolecular decay of substituted Criegee intermediates to OH, including tunneling effects at atmospheric temperatures. These rate data, resolved for specific Criegee structures such as methyl vinyl ketone oxide, are critical for accurately representing HO_*x*_ radical budgets in isoprene-rich environments.

Building on this Criegee-focused work, Stone and co-workers have provided high-precision bimolecular rate coefficients for key Criegee loss processes. Measurements of CH_2_OO + NO_2_ kinetics over 242–353 K revealed an unexpected negative temperature dependence and rates substantially different from prior theory, prompting re-evaluation of mechanism parameters.^76^ Parallel conformer-specific studies of CH_3_CHOO reactions with water monomers and dimers quantified major atmospheric sinks for syn- and anticonformers over 260–350 K, extending the kinetic database beyond the narrow temperature ranges previously available.^77^ These results enable improved representation of Criegee removal pathways in near-explicit mechanisms such as the MCM and global chemistry–transport models like GEOS-Chem, reducing uncertainty in HO_*x*_ and secondary organic aerosol predictions.

Together, these developments illustrate how advances in automated mechanism generation and model–measurement integration are expanding the predictive skill of atmospheric chemistry models, enabling them to capture both canonical and previously overlooked reaction pathways across gas, multiphase, and heterogeneous domains.

**Figure 3:**
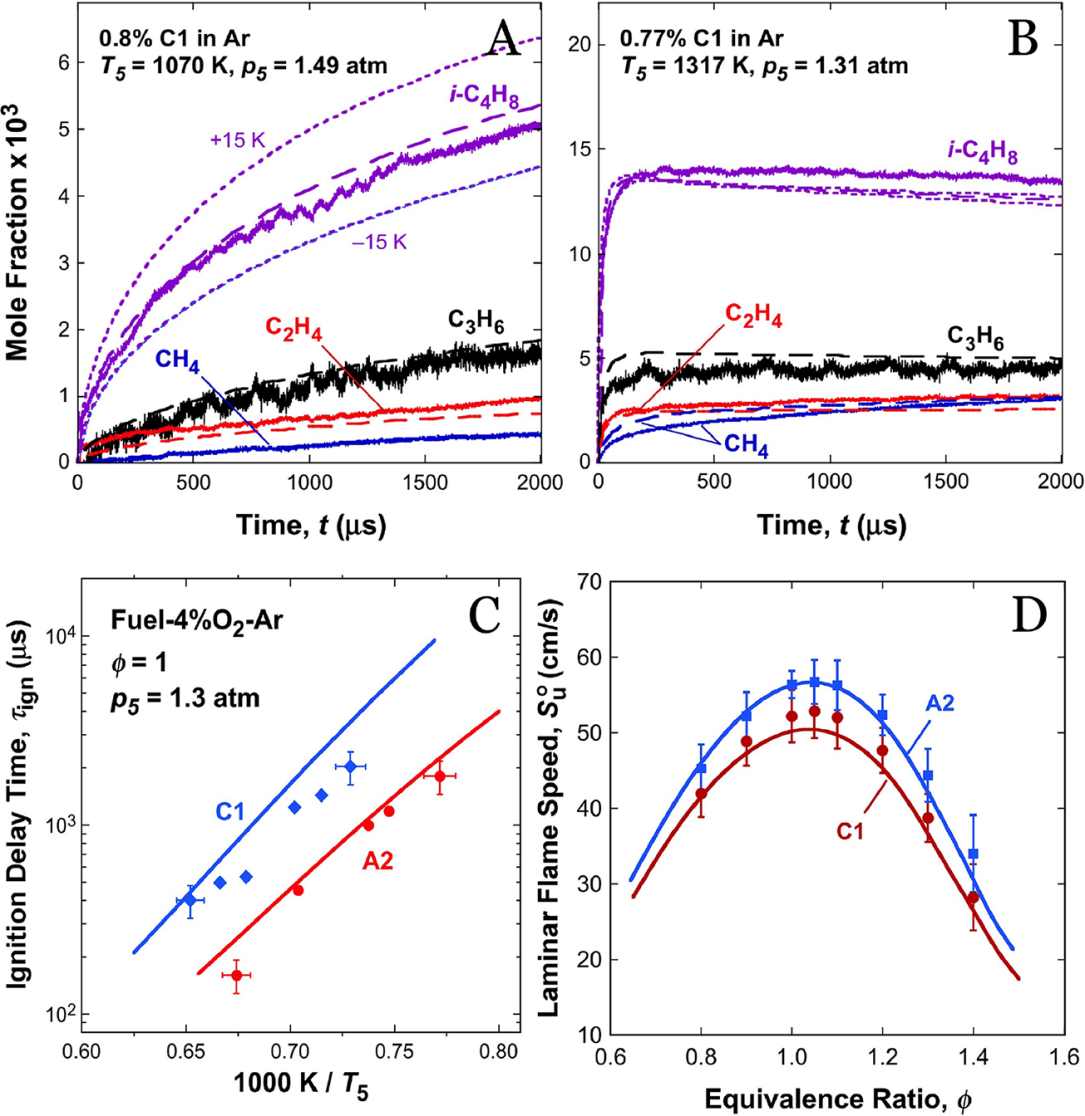
Experimental validation of a chemical kinetic model. (A) Experimental (solid lines) and computed (long dashed lines) species time histories during the pyrolysis of an alcohol-to-jet fuel mixture in a shock tube under postreflected shock temperature and pressure of 1371 K and 1.31 atm, respectively. (B) Experimental (symbols) and computed (lines) species concentrations during oxidative pyrolysis of an alcohol-to-jet fuel mixture in a flow reactor at 1030 K, *p* = 1 atm, input fuel concentration of 305 ppm, equivalence ratio of 1. (C) Experimental (symbols) and computed (lines) ignition delay times of Jet A and an alcohol-to-jet fuel mixture in 4% O_2_ diluted by Ar. (D) Measured (symbols) and simulated (lines) laminar flame speeds of Jet A (in red) and an alcohol-to-jet fuel mixture (in blue) at 1 atm. This figure was adapted from ref 42. Copyright Elsevier (2018).

### 2.4 Electrochemical Reaction Kinetics

In electrocatalysis, predictive modeling demands theoretical frameworks that quantitatively translate atomistic descriptions of interfacial reaction pathways into experimentally measurable performance metrics such as current–potential behavior, selectivity, and stability. Among these, proton-coupled electron transfer (PCET) provides a unifying description for the rate- and selectivity-determining steps across diverse electrocatalytic reactions, including CO_2_ reduction, oxygen and hydrogen evolution reactions, oxygen reduction, and nitrogen reduction. Theoretical developments^16,78–81^ have yielded nonadiabatic rate constant expressions for both homogeneous and heterogeneous PCET in adiabatic, nonadiabatic, and solvent-controlled regimes. These formulations incorporate quantum treatment of the proton, excited vibronic states, and interfacial effects such as the electric double layer and solvent reorganization. They quantitatively reproduce experimental observables, including potential-dependent kinetic isotope effects and nonideal Tafel slopes,^82^ and their outputs can serve directly as inputs to electrochemical microkinetic models and voltammogram simulations.

Electrochemical kinetic modeling extends beyond low-temperature molecular systems. In solid oxide electrolysis cells (SOECs), high-temperature (≳800 °C) kinetics govern the coelectrolysis of CO_2_ and H_2_O to produce syngas and higher hydrocarbons. For example, Nb- and Sr-doped LaFeO_3_ fuel electrodes exhibit high current densities and long-term stability in CO_2_/H_2_O feeds, with measured rates and Faradaic efficiencies providing benchmarks for microkinetic and reactor-scale SOEC models.^83^

At the system scale, kinetic parameters strongly influence process feasibility. Techno-economic analysis of CO_2_ electroreduction to ethylene^84^ shows that even with optimal reaction kinetics, selectivity and energy efficiency must meet stringent targets for economic viability under current market conditions. This underscores the importance of coupling accurate microkinetic modeling with realistic process evaluation.

These examples demonstrate that electrochemical reaction kinetics, when informed by rigorous mechanistic theory and integrated with validated computational models, can predict potential–current relationships, selectivity trends, and stability metrics with increasing accuracy, enabling rational catalyst and device design across a wide range of electrochemical technologies.

### 2.5 Biomass Conversion Processes

Chemical kinetic modeling of biomass pyrolysis and gasification has matured to the point where detailed mechanisms can quantitatively reproduce measured product distributions, mass-loss profiles, and devolatilization rates across a wide range of feedstocks and operating conditions. Frameworks such as the chemical percolation devolatilization (CPD) family, its biomass-specific extensions (Bio-CPD), and the CRECK biomass mechanisms resolve the parallel and sequential decomposition of cellulose, hemicellulose, lignin, and extractives into char, condensable organics (tar or bio-oil), and permanent gases.^85–87^ These models employ reference constituent characterization, reaction-family rate rules, and thermochemistry-based analogies to parametrize devolatilization chemistry, and have been validated against drop-tube, thermogravimetric analysis (TGA), and fast-pyrolysis reactor data.

The CPD and Bio-CPD approaches simulate primary and secondary decomposition with sufficient fidelity to match major product yields within ∼10% of experimental values for both hardwoods and softwoods, while also capturing the release kinetics and gas-phase oxidation of the volatiles.^85,86^ The CRECK biomass mechanism extends these capabilities by coupling the devolatilization stage to a detailed gas-phase oxidation network originally developed for hydrocarbon fuels, enabling predictive reactor-scale simulations of pyrolysis, gasification, and combustion of lignocellulosic materials.^87^ Such integrated descriptions are essential for translating particle-scale chemistry into reactor-scale performance predictions.

Beyond comprehensive full-detail models, recent developments have focused on complexity reduction and coupling with transport models. Martins et al. applied an error-based species and reaction elimination strategy to a 134-species, 4533-reaction secondary gas-phase mechanism, producing a reduced model with 89 species and 476 reactions that preserved the accuracy of gas composition and release rate predictions while halving computational cost.^88^ Equivalent reactor network (ERN) modeling has been used to represent fluidized-bed gasifiers, coupling equilibrium and perfectly stirred reactor (PSR) modules with plug-flow reactor (PFR) descriptions to predict gas yields, tar formation, and optimal equivalence ratios for operation.^89^

Multiscale integration remains a central theme. Reviews by Kostetskyy and Broadbelt^90^ and Vikram et al.^91^ emphasize that accurate biomass conversion modeling requires linking distributed kinetic models with CFD to resolve the hydrodynamic–reaction coupling in particle-laden reactive flows, and point to emerging tools such as ANN-based surrogates and UQ frameworks to extend predictive reach while controlling computational cost. These efforts underscore that, when carefully parametrized and validated, biomass pyrolysis and gasification kinetic models can serve as robust predictive tools for reactor design, optimization, and scale-up.

### 2.6 Enablers

Across several kinetic modeling domains, enabling infrastructure has transformed what used to be months of manual calculations into streamlined, automated workflows capable of producing refined predictive chemical kinetic models (Figure 2). These tools fall broadly into three complementary categories: automated model generation, automated model refinement, and automated model development, each addressing a different stage of the model-building process.

Automated model generation frameworks, such as RMG^4,47^ and Genesys,^7,50^ systematically construct large-scale reaction networks by applying reaction family templates, thermochemical estimation schemes, and kinetic rate rules to a user-defined chemical system. RMG has evolved from a combustion-focused gas-phase tool into a multiphase platform capable of handling heterogeneous catalysis and liquid-phase chemistry, with implementations underway for electrochemistry. It uses a flux-based algorithm to iteratively enlarge the model until a target termination criterion is met, estimating missing parameters with group additivity methods and rate rules validated by community-curated databases. Genesys employs a graph-based approach to exhaustively enumerate reaction possibilities from given reactants and rule-based species selection criteria. Its heterogeneous extension, Genesys-Cat, integrates DFT-derived thermochemistry and kinetic data, optionally refined via Bayesian optimization, to deliver predictive microkinetic models.

Once the (initial) mechanism is generated, a different class of tools automates the calculation of selected high-accuracy thermochemistry and kinetic rate coefficients, refining database estimates of key parameters with first-principles predictions. EStokTP^92^ focuses on large sets of bimolecular reactions, providing an integrated environment for both electronic structure calculations and Transition State Theory (TST)-based master equation (ME) kinetics. It automates conformer identification, torsional treatments (including multidimensional hindered rotors), and variational TST (VTST). Its robust job handling, failure recovery, and support for abstraction, addition, isomerization, and β-scission reactions make it practical to compute a large number of rate coefficients without significant manual intervention. AutoTST^93^ automates rate calculations for selected reaction families common in combustion chemistry, currently hydrogen abstraction, disproportionation, and intramolecular H-migration. It incorporates systematic conformer searches, vibrational analyses to verify transition states (TSs), accurate symmetry number determination, and hindered rotor corrections. Benchmarks show that AutoTST can locate valid TS geometries for over 90% of test cases and return reliable rate coefficients for the majority, which can then directly replace estimated rates in detailed kinetic models. Chemical Trajectory Analyzer (ChemTraYzer)^94,95^ uses reactive molecular dynamics (rMD) simulations, allowing for the efficient and parallel investigation of complex mechanisms. It automatically combines them with quantum mechanical optimizations for species and transition states to generate thermodynamic data and reaction rate coefficients. KinBot^96,97^ focuses on unimolecular reaction networks, automatically exploring the potential energy surface (PES) of an input species, locating relevant energy wells and TSs, and outputting complete reaction networks. It has been demonstrated in combustion and atmospheric chemistry contexts for species undergoing molecular weight growth, autoxidation, and other complex rearrangements. AutoMech^98^ is a modular suite for large-scale, high-level computational thermochemistry and kinetics. It implements a complete workflow from parsing a mechanism and species dictionary, to Monte Carlo conformer searches, IRC and prereactive van der Waals complexes, TS guesses, high-level quantum chemical property calculations, ME simulations, and temperature/pressure dependence rate coefficient fitting. Each of its libraries, AutoChem, AutoIO, AutoFile, MechDriver, and MechAnalyzer, can be used independently, but together they enable end-to-end kinetic model parametrization. ARC^99^ (Automated Rate Calculator) targets the direct computation of thermodynamic properties and reaction rate coefficients from simple 2D species descriptors such as SMILES, InChI, or RMG adjacency lists. It performs a conformer search, atom-maps reactions in 3D, searches for TSs, and automatically submits and monitors electronic structure jobs on user-defined HPC resources. It troubleshoots failed jobs, and finally processes outputs into NASA polynomials and Arrhenius expressions, and integrates with RMG. All of the above tools are open-source.

Higher-level orchestration tools link model generation and refinement into a single iterative model development workflow. T3,^100^ The Tandem Tool for automated chemical kinetic model development, is an open-source tool that integrates RMG for network generation with ARC for parameter refinement in an automated loop. At each iteration, T3 performs sensitivity analysis to identify the top thermodynamic properties, rate coefficients, or zero-point energies within a PES influencing target observables. Those parameters are recalculated at higher fidelity by executing ARC, the model is regenerated, and the process repeats until convergence or a user-defined iteration limit is reached. This approach focuses computational resources on the parameters with the greatest impact, accelerating convergence toward well-validated and predictive models.

Beyond these core automation categories, complementary infrastructure supports simulation, data exchange, and UQ. Cantera^101^ provides a robust, open platform for simulating chemically reacting flows, integrating detailed mechanisms into reactor, flame, and transport models. Data standards such as ChemKED^102^ enable machine-readable storage and exchange of experimental ignition, species time-history, and other kinetics data, facilitating direct model-experiment integration. Advanced UQ methods, including local and global sensitivity analysis, Bayesian parameter estimation, and polynomial chaos expansions, are increasingly embedded in these workflows to produce decision-grade, transparent kinetic models.^25^ ML augments these efforts by accelerating property prediction,^28,30,103,104^ automating pathway discovery, and inferring rate coefficients directly from experimental time-series data.^33^

Data fusion enhances predictive modeling by integrating multisource analytical signals through three primary hierarchical strategies: low-level fusion of raw data, midlevel fusion of extracted features, and high-level fusion of independent model outcomes. The effectiveness of these methodologies relies on characterizing the underlying data structure to determine the appropriate preprocessing and linkage between sample and variable modes.^105^ This practice allows models to continuously update as new experimental or simulation data become available, reducing uncertainty and improving predictive accuracy across a wide range of conditions. Indeed, the lack of high-quality kinetic data has long been a key obstacle. Hence, efforts to generate new data sets and develop data-efficient ML techniques^27^ are pivotal for advancing kinetics modeling. This practice should be done cautiously, preserving physics and provenance to keep models realistic and predictive.

Literature mining offers promising avenues for augmenting mechanistic models with data-driven insights. Natural language processing and text-mining tools can automatically extract reaction information and kinetic parameters from the wealth of scientific literature.^106,107^ Such capabilities can rapidly populate models with known reaction pathways and rate coefficients that would otherwise require manual curation. Caution, of course, must be practiced to verify the accuracy and context of the extracted data. Together, the infusion of ML, data fusion, and literature-derived knowledge into the modeling workflow holds the promise of hybrid models that are both physics-grounded and continuously informed by data. These developments are a key step toward the autonomous, closed-loop modeling paradigm envisioned for sustainable chemical kinetics.

Taken together, this ecosystem of automated generation, refinement, and development tools, combined with open simulation platforms, data standards, and UQ frameworks, now allows the community to progress from an initial chemistry concept to a refined, uncertainty-quantified predictive model with unprecedented speed, scope, and reproducibility.

Finally, democratization requires a cultural shift regarding data stewardship and software interoperability. Currently, the field suffers from a lack of standardized ’round-robin’ benchmarks, leading to scenarios where different software packages yield discrepant results for identical inputs, a challenge well-known in the CFD and quantum chemistry communities. To resolve this, we need not just shared files, but shared infrastructure for metadata and rigorous cross-validation. The goal is to move from isolated workflows to a cohesive ecosystem where discrepancies are automatically flagged and are attempted to be resolved, ensuring that ’reproducibility’ means obtaining the same answer regardless of the solver employed.

### 2.7 Summary

Across combustion, catalysis, atmospheric chemistry, electrochemistry, and biomass conversion, detailed kinetic models have reached a level of maturity where they can reproduce key experimental observables such as flame speeds, ignition delays, catalytic turnover rates, pollutant concentration trends, electrochemical current–potential curves, and pyrolysis yields, within experimental uncertainty when applied in validated regimes. In each case, predictive capability arises from a combination of physically sound reaction networks, accurate thermochemistry, and refined rate coefficients. These successes are increasingly underpinned by enabling infrastructure: automated mechanism generation and refinement, high-fidelity thermochemical and kinetic workflows, and open simulation platforms.

The cumulative evidence from these domains demonstrates that, where the chemistry is well-characterized, kinetic modeling can function as a quantitatively predictive tool for both interpreting existing data and forecasting behavior under new conditions. As computational methods, experimental diagnostics, and data integration strategies continue to advance, the scope of reliable kinetic predictions is expected to expand to more complex, multiphase, and strongly coupled systems, broadening the impact of kinetic modeling in science, engineering, and technology development.

When the chemistry is well-characterized, kinetic modeling can now predict key observables across diverse domains with quantitative accuracy, providing a reliable tool for both interpreting data and forecasting behavior.

## 3 What Is Still Hard

Despite the tremendous progress achieved in kinetic modeling, fundamental challenges remain, and many of them grow sharper as we tackle more complex systems (Figure 4). The domains of applicability for predictive chemical kinetics should be thought of as a continuum rather than a strict dichotomy: the practical boundary between “can do well” and “still hard” is often set by the level of idealization in the underlying models and data. Gas-phase reactions of small molecules under ideal conditions can now be modeled with high fidelity. However, much of chemistry does not occur in such well-behaved environments. Real systems involve larger molecules, condensed phases, complex and evolving surfaces, or extreme conditions that strain our current models. Even for some seemingly ”simple” small molecules containing heteroatoms, predictive modeling is still imperfect.

**Figure 4:**
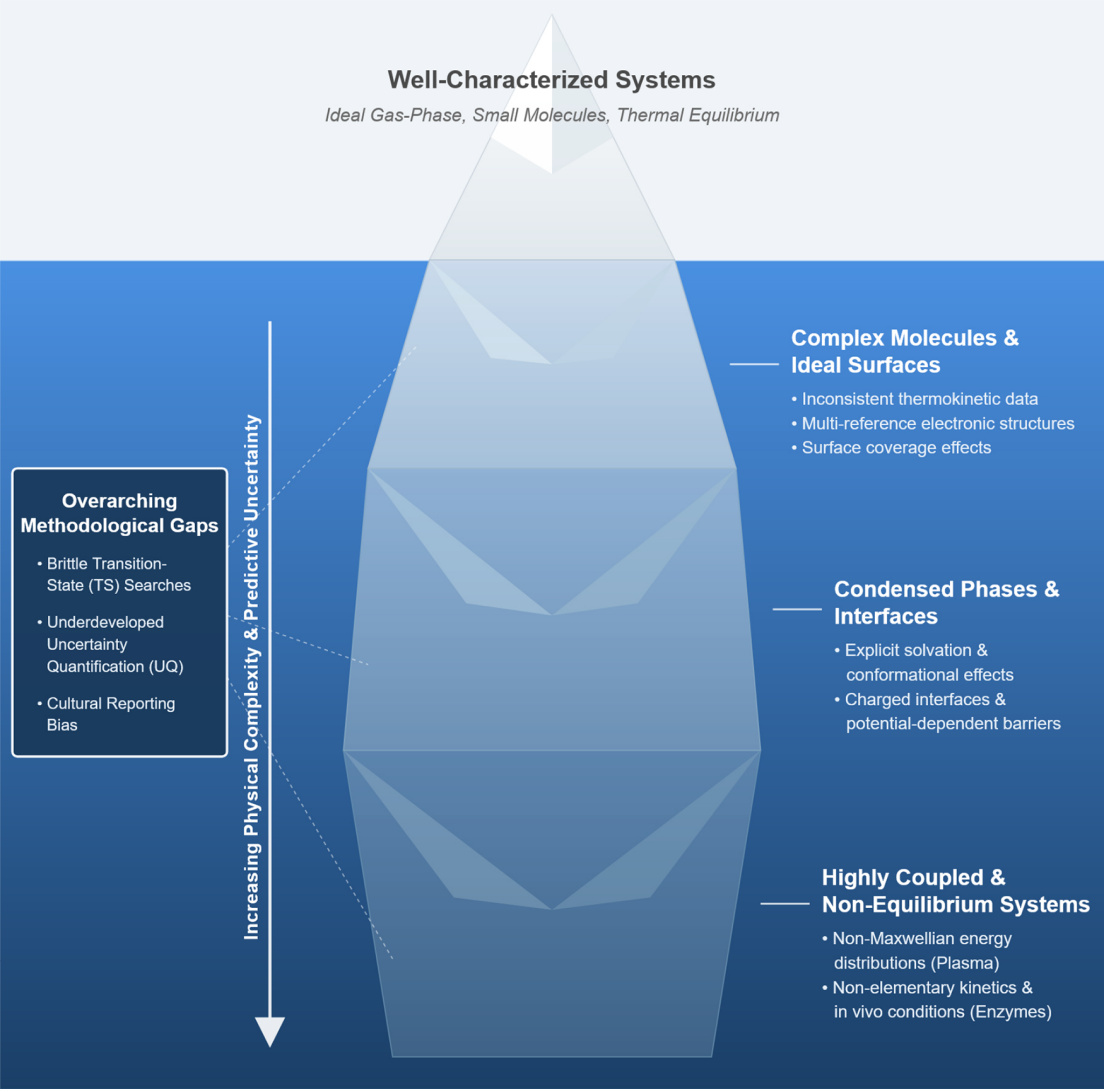
Iceberg of Complexity, illustrating the layered challenges that emerge in predictive chemical kinetic modeling as systems move from well-characterized ideal conditions to complex, condensed, and nonequilibrium environments.

As discussed in Section 2, there are several examples where elementary-step kinetic models (with parameters derived from a mix of quantum chemistry calculations and single-reaction or single-molecule experiments) have been successfully combined with lumped submechanisms derived from fitting experimental final product distributions to create useful kinetic models. To date, this sort of combination has been done by experts in each subfield, who made many decisions about how to use the experimental data and how to structure the final models. Further research is needed into how best to combine disparate data, and how to determine the confidence limits on the resulting kinetic models, and under which conditions they are valid. In the following subsections, we outline several areas where kinetic modeling remains particularly hard and additional work is welcome.

Despite two decades of progress, three constraints recur across domains. (i) Information: kinetic/thermochemical data remain sparse, biased toward easy conditions, and uneven across elements (notably heteroatoms in complex organics), forcing heuristics and local calibration. (ii) Physics: many targets involve multireference character, non-Born–Oppenheimer effects, field/phase coupling (solvation, electrochemical double layers, radiation), or distributed active sites, regimes where scalable, size-extensive approximations are immature. (iii) Algorithms: today’s rule-based generators and TS workflows assume gas-phase-like topologies; they struggle with conformational explosion, and multiple spin surfaces, so missing pieces are silently backfilled by estimates with poorly quantified uncertainty. Together, these factors yield pipelines that are unreliable in some regimes. Emerging directions such as automated microsolvation, ML surrogates for PES exploration and thermodynamic property calculations, and experiment-in-the-loop active learning coupled to rigorous UQ, directly target some of these failure modes.

### 3.1 Small Heteroatom Molecules

While hydrogen and simple hydrocarbons are well-understood, small heteroatom-containing molecules such as ammonia (NH_3_) and hydrogen sulfide (H_2_S) remain challenging despite decades of study. For NH_3_, recent reviews of modern oxidation mechanisms reveal significant inconsistencies in thermodynamic data, multiorder-of-magnitude disagreements in key rate coefficients, leading to the “many-model” problem.^108^ While many detailed mechanisms exist, none can capture all combustion behaviors across all conditions.^109^ These discrepancies limit predictive reliability and highlight the need for standardized, traceable thermokinetic parameters. Moreover, cross-reactions between nitrogen- and carbon-containing species are still not entirely resolved.^40^

Sulfur systems face analogous challenges. In H_2_S combustion, the primary H_2_S → SO_2_ pathway is generally well captured, but when combusted with a carbon fuel kinetically sensitive intermediates such as COS, CS_2_, mercaptans, and sulfur-containing PAHs, are often poorly predicted. This stems from sparse and uncertain kinetic data, strong coupling between sulfur and hydrocarbon chemistry, and the need to model multispin-state potential energy surfaces for key reactions.^110^ Industrially relevant conditions, such as those in Claus units and sulfur recovery tail-gas treatment, further complicate validation due to multiphase effects, turbulence, and heat transfer. High-quality measurements, consistent parameter evaluation, and expanded quantum–experimental data sets are essential to improve predictive capability for these small but chemically complex systems.

Although algorithmic frameworks are now available to automatically generate and filter the complex resonance structures inherent to nitrogen- and sulfur-containing species,^111^ accurate thermodynamic and kinetic parametrization remains a bottleneck. Current automated tools are struggling because their underlying estimation schemes, initially designed for neutral oxigenated hydrocarbons, often lack the fidelity to handle the partial charges and zwitterionic character prevalent in these heteroatomic systems. Moreover, the multireference (MR) electronic character typical of N- and S-oxide intermediates complicates standard high-throughput quantum refinement, as well as pressure-dependent rate computations due to size-extensivity limitations of many MR methods. Moreover, pressure-dependent networks of oxygenated heteroatom species frequently traverse exotic pathways that lie outside the domain of established reaction templates or involve intersystem crossing of spin surfaces.

A further complication is the inadvertent generation of “nonphysical” species—artifacts that emerge when automated tools apply reaction templates to explore vast chemical spaces. While these species often satisfy standard valency constraints and appear chemically plausible, rigorous PES exploration reveals they lack a stable energy well, dissociating barrierlessly into lower-energy fragments.^112^ If undetected, these pseudospecies can become embedded in pressure-dependent reaction networks, acting as artificial bottlenecks that distort computed rate coefficients by orders of magnitude, as demonstrated in recent case studies of diazenyl peroxide intermediates in nitrogen combustion systems.^112^

### 3.2 Oxygenated Fuels

While predictive combustion modeling of small hydrocarbons has reached high maturity, oxygenated fuels remain more challenging to model consistently across all operating conditions. Even for relatively simple species like dimethyl ether (DME), no single mechanism captures all observables accurately. Hanson and co-workers^113^ has shown that the model providing the best ignition delay time (IDT) predictions performs poorly for laminar flame speeds (SL), underscoring the challenge of developing mechanisms transferable between autoignition and flame-propagation regimes. More generally, systematic data sets of IDTs for small hydrocarbons at elevated pressures provide uniform constraints for refining shared core submechanisms, yet similar coverage for oxygenates is still lacking.^114^ For smaller oxygenates such as formic acid (HOCHO), automated mechanism generation can yield first-iteration models performing comparably to hand-tuned mechanisms, but unresolved issues, most notably the pressure-dependent branching ratio (HOCHO *⇌* CO_2_ + H_2_) and dehydration (HOCHO *⇌* CO + H_2_O), persist.^115^ These uncertainties in the CH_2_O_2_ PES propagate into larger oxygenated-fuel models.

At a broader level, integrating oxygenate chemistry into multifuel combustion mechanisms is hindered by inconsistencies in kinetic submodels, incomplete pressure-dependent networks, and the scarcity of speciation data under realistic engine conditions. Addressing these gaps will require simultaneous validation of multiple observables (IDT, SL, extinction strain rate, and speciation profiles) across wide temperature/pressure ranges, with a consistent treatment of pressure-dependent branching and cross-reaction chemistry.

### 3.3 Heterogeneous Catalysis

Heterogeneous catalysis remains a major frontier of uncertainty in kinetic modeling. While modern theory and computation allow detailed simulations on idealized crystalline surfaces, real catalysts present much greater complexity. Practical catalysts are often nanoscale, multicomponent materials with a variety of facets, defects, and site types, leading to broad distributions of binding energies rather than a single ”Sabatier-optimal” value.^116^ Here we move from well-characterized single-facet metals to fluxional, defect-rich supports, which break scaling relations. Such surface heterogeneity produces nonideal adsorption isotherms and can even break the linear free-energy scaling relations that underpin many microkinetic models. Moreover, adsorbate–adsorbate interactions (especially among strongly bound species) can introduce coverage-dependent rate effects that simple mean-field models struggle to capture. In short, the local catalyst environment – from atomic geometry to adsorbate coverage – can strongly skew reaction rates in ways that are not easily parametrized by current mean-field approaches.

Another challenge is the dynamic nature of working catalysts. Under reaction conditions, surfaces are not static: adsorbates can induce reconstructions or particle reshaping, creating new active sites or deactivating others over time. Standard microkinetic modeling usually assumes a fixed lattice of sites, so it cannot account for such time-evolving site ensembles. Detailed case studies have shown that reactants can restructure a catalyst (for example, CO-induced cluster formation on Cu) and alter its activity, requiring advanced methods like lattice Monte Carlo or molecular dynamics to simulate these effects.^117^ Because fully explicit treatments of surface fluxionality are computationally expensive, most kinetic models of catalysis still rely on mean-field approximations on idealized surfaces, with coverage effects folded in via simplistic corrections. This often necessitates empirical tuning, adjusting DFT-derived parameters to fit experimental data, which compromises the model’s transferability and predictive power. At present we are usually limited to using DFT rather than the higher level coupled-cluster or MR methods we can use in gas phase, leading to less accurate predictions. Achieving truly predictive microkinetic models for heterogeneous catalysis will require embracing this complexity: accounting for multisite reaction mechanisms and dynamic surface changes. Each of these aspects remains an open challenge, and addressing them is crucial for reliable simulation of real-world catalytic systems.

### 3.4 Liquid-Phase Kinetics

Liquid-phase reaction kinetics present a major challenge compared to gas-phase systems. Solvent effects, including intermolecular hydrogen bonding, dielectric stabilization, conformational changes, and additional entropy effects, can dramatically affect reaction rates. However, these factors are difficult to capture with traditional quantum chemistry alone. Even a small error in estimating a solvation-influenced energy barrier height can lead to orders-of-magnitude errors in the predicted rate coefficient, especially at relatively low temperatures. Accurate kinetic modeling in solution is therefore inherently complex.

A variety of computational strategies exist to include solvation effects, each with successes and limitations. Common continuum solvation models, most notably polarizable continuum model (PCM)^118^ and continuum solvation model density (SMD)^119^ treat the solvent as a homogeneous dielectric medium. These methods are popular for their reasonable accuracy versus cost trade-off, but they rely on approximations that sometimes break down, e.g., for multicomponent solvent systems. In fact, standard quantum continuum models typically assume a single pure solvent at a fixed temperature, limiting their applicability in more complex conditions. Their performance on reaction energetics can be inconsistent,^120^ for example, a continuum model may underestimate stabilization in a nonpolar solvent or fail to capture specific solute–solvent interactions, leading to significant errors in rate coefficient predictions. More advanced implicit schemes like COSMO-RS (Conductor-like Screening Model for Real Solvents) introduce statistical thermodynamics of solvent effects and can handle mixtures and temperature dependence to some extent.^121,122^ COSMO-RS and its variants often improve the accuracy for many neutral solutes, and can be surprisingly accurate for kinetic solvent effects for reactions where all reactants and products are neutral.^123^ Errors in computed solvation energies of ionic species are much larger, even after empirical functional group type corrections,^124^ though if the solvation energy of an ion in one solvent can be determined, its solvation in other solvents can be predicted fairly accurately.^125^

On the other end of the spectrum, explicit solvent simulations (QM/MM or molecular dynamics with many solvent molecules) can, in principle, capture detailed solute–solvent interactions. However, these are extremely computationally expensive, requiring thousands of solvent molecules and extensive sampling, and thus are impractical for high-throughput kinetics studies.^122^ Hybrid approaches have been proposed to bridge the gap; for instance, adding a few explicit solvent molecules around the reactive site (a cluster-continuum model) can significantly improve predicted solvation energies for strongly interacting systems, compared to a pure continuum model.^126^ Still, such microsolvation techniques only incrementally extend the capability and remain too costly for routine use across an entire reaction network.

In solution, molecules can adopt different conformers than in the gas phase due to solvent stabilization. This adds another layer of complexity: one must consider multiple conformations of reactants and TSs to obtain reliable reaction free energies. In fact, the preferred conformations determined for a molecule can change significantly depending on whether the calculations were performed in the gas phase or assuming implicit or fully explicit solvent conditions.^127^ Therefore, extensive conformer searches (potentially using molecular dynamics or advanced sampling) may be required to find the relevant low-energy structures in solution. This is a computational bottleneck, especially for large, flexible molecules, as each conformer might need separate quantum evaluations with solvation. Entropic contributions in the liquid phase also differ from those in the gas phase; for example, the entropy loss upon bimolecular association is partly offset by solvent caging and reduced translational freedom, and viscous diffusion can limit reaction rates in extreme cases. Capturing these effects demands free-energy calculations (including solvation and mixing entropy), which are far more involved than a standard gas-phase kinetic calculation.

Recently, Türtscher and Reiher^128^ introduced automated microsolvation for minimum-energy path construction in solution, a significant step toward automation in solvated TS modeling. Their protocol first performs TS optimization in a partially solvated QM/MM environment, then systematically identifies and retains only the active solvent molecules that directly participate in the reaction’s key normal mode, yielding a reduced, computationally tractable microsolvation model. Remaining solvent effects are captured via continuum models, and cavity entropy corrections are applied to approximate free-energy contributions. This hybrid workflow is a step in the right direction toward efficient, high-throughput microkinetic modeling in solution.

A different approach that shows promise has been explored by Serse et al.,^129^ as well as others,^130^ and consists of determining rate coefficients from MD simulations performed using enhanced sampling and metadynamics. The strength of this methodology lies in the use of reactive force fields to determine free energy profiles for the investigated reaction channels. Rate coefficients can then be determined by evaluating the unbiased net reaction flux from reactants to products.^131^ While these approaches provide a rigorous framework for the estimation of the conformational sampling, as well as a context for the discovery of reaction pathways, their accuracy is directly related to that of the used reactive force field. The construction of force fields with ab initio level accuracies for reactions taking place in solution, but not only, is therefore an extremely active research subject, with significant progress being made in combining evaluation of energies through first-principle calculations and construction of PESs using ML methodologies.^132^ Developing an automated, reliable pipeline for liquid-phase kinetics remains an open challenge. Ideally, such a pipeline would integrate robust conformer sampling in solution, accurate solvation free energies for reactants and TSs, and efficient TS searching under solvent influence. At present, no such end-to-end solution exists.

### 3.5 Electrochemical and Solid-State Kinetics

Electrochemical and solid-state reaction systems, such as battery electrodes, fuel cells, electrolyzers, corrosion processes, and photoelectrocatalytic interfaces, introduce additional complexities beyond those in homogeneous kinetics. Reactions at solid–electrolyte interfaces are influenced by the catalyst surface as well as by the interaction of charged species, electric fields, and the surrounding medium.^133^ Factors like charge transfer at the electrode, interfacial electric double layers, solvent or solid electrolyte structure, and even defect sites in the solid can all significantly alter reaction pathways. For example, the electrochemical interface is inherently complex: the electrode potential, the hydrogen-bond network of the adjacent solvent, the coverage of intermediates, and the dynamic reorganization of the interface collectively affect reaction rates and selectivity. A major theoretical challenge is thus to accurately describe this charged interface between an electrolyte and a conductor, which traditional models handle only approximately.^134^ Common approaches like implicit continuum solvation tend to place counter-charges unrealistically and cannot capture local variations in the interfacial field. In practice, solid surfaces may also reconstruct or form transient states under bias (e.g., as ions insert or potentials change), further complicating first-principles modeling.^134^

Another core difficulty lies in potential-dependent reaction energetics and kinetics. Unlike neutral gas-surface reactions, electrochemical reaction steps (often proton- or electron-transfer steps) have free energies and barriers that vary with applied electrode potential and with the distribution of charge in the double layer. Capturing this in first-principles calculations is nontrivial. Applying a constant electrode potential in simulations requires special methods, and standard DFT at fixed charge often misses the correct potential dependence.^135^ As a result, microkinetic models typically must incorporate the voltage dependence empirically (e.g., via Butler–Volmer-type transfer coefficients), which introduces uncertainty.^135^ Indeed, the reaction rate sensitivity to electrode potential and solvation is strong, and calculating how a barrier changes with potential is difficult, often compromising purely predictive modeling. Recent studies using grand-canonical DFT have shown that electrochemical barriers can respond nonlinearly to the applied bias. They are primarily governed by the surface’s excess charge (related to its work function), a fundamentally different controlling factor than in conventional catalysis.^136^ This means that intuitive linear free-energy relationships or gas-phase descriptors may not hold once an external potential and charged interface are involved.

Finally, there is a lack of robust, transferable descriptors and benchmark data for these complex systems. In “simpler” systems, such as heterogeneous catalysis, one can often correlate activity to a few key energetics (like adsorption energies). Still, in electrochemical or solid-state contexts the relevant energetics depend on multiple coupled variables (potential, solvent, ion identity, defects, etc.). No single descriptor easily captures all these effects.^133^ Factors such as the solvent environment, pH, and even DFT setup can significantly change calculated reaction energetics, underscoring the need to consider multiple factors rather than any one “universal” descriptor.^133^ Conventional scaling relations (e.g., Sabatier volcano plots based on one adsorbate’s Δ*G*) often break down for electrochemical steps under working conditions, due to solvent dipole interactions and field effects that alter reaction kinetics beyond what a single thermodynamic parameter can predict. Moreover, experimental benchmarks for elementary steps at electrochemical interfaces are limited, since it is challenging to directly probe interface structure and reaction rates at operating potentials. This scarcity of direct data means that theoretical models cannot be rigorously benchmarked for all conditions. Consequently, many kinetic models in batteries, fuel cells, and related solid-state systems remain semiempirical and case-specific; they rely on fitted parameters (e.g., Tafel slopes, exchange currents) tuned to particular materials or conditions, which limits their transferability to new systems.

### 3.6 Plasma Systems

Plasma chemistry sits at the extreme end of kinetic modeling complexity. Unlike purely thermal systems, plasmas generate and sustain a wide variety of species (ions, electrons, radicals, and electronically and vibrationally excited neutrals) under highly nonequilibrium conditions. These populations interact through electron-impact reactions, radiative processes, and ion–neutral or ion–surface collisions, often with reaction pathways that are absent in conventional chemistry.^17^ Capturing these effects in a chemical kinetic framework requires simultaneous treatment of plasma physics, chemical reaction networks, and transport phenomena.

One of the central challenges is that there is no widely adopted “standard” plasma mechanism analogous to those in combustion or atmospheric chemistry. Each application, whether plasma-assisted catalysis, atmospheric-pressure plasma processing, or space re-entry flow, typically relies on a custom-built reaction set, assembled from disparate sources and tuned to specific operating conditions.^137^ Transferability between systems is limited because rate coefficients for electron-impact excitation, ionization, and dissociation depend strongly on the electron distribution function, which is often highly non-Maxwellian and sensitive to the local plasma environment. Unlike in purely thermal systems, the behavior cannot be captured by simple parametrizations in terms of just a few variables such as pressure, temperature, and gas composition.^138^

Radiative transport adds yet another layer of complexity. In many plasma systems, particularly those used in materials processing or re-entry environments, emission and reabsorption of photons significantly affect both energy balance and species populations. Modeling these effects accurately demands spectral radiation models coupled to the plasma kinetics.

Overall, plasma systems challenge the limits of chemical kinetic modeling because they require consistent, simultaneous resolution of chemical and physical nonequilibrium processes across a wide range of time and length scales. The absence of standardized, transferable plasma reaction mechanisms, the difficulty of predicting Electron Energy Distribution Function (EEDF) dependent rate coefficients, and the computational cost of fully coupled multiphysics simulations are persistent barriers. Progress will likely depend on developing modular, hybrid modeling frameworks that can selectively apply high-fidelity plasma physics where needed while retaining computationally efficient chemistry solvers elsewhere.

### 3.7 Enzymatic Reactions

Modeling the chemistry of an enzyme’s active site often requires quantum mechanical methods to capture bond-breaking, electron transfer, and other catalytic electronic effects. Hybrid QM/MM approaches treat the reactive center quantum-mechanically and the rest of the protein classically.^139^ These computations are still time-limited, and capturing complex multistep reaction mechanisms, transition states, and dynamic allostery in silico remains difficult. These quantum-level challenges mean that active-site kinetics often rely on simplified models or precomputed potential energy surfaces, which may not fully represent the enzyme’s behavior under cellular conditions.

At the larger scale of pathways and metabolic networks, modeling enzymatic reaction kinetics in solution introduces a different set of challenges. Building mechanistic kinetic models requires a large number of enzyme-specific parameters (rate constants, *K*_m_, *k*_cat_, etc.), but comprehensive experimental data are usually lacking.^140^ The scarcity and inconsistency of kinetic data, compounded by differences between in vitro measurements and in vivo conditions, make it difficult to parametrize models reliably.

Unlike simple chemical reactions, enzymatic kinetics are usually nonelementary, arising from multistep mechanisms and enzyme–substrate complexes. A single enzyme-catalyzed reaction may involve several intermediates, conformational changes, or multiple substrates, yielding rate laws that are more complex than mass-action kinetics. Modelers often resort to simplified rate laws (e.g., Michaelis–Menten or Hill equations) to reduce complexity. Michaelis–Menten kinetics, for instance, lump a complex mechanism into two apparent parameters (*V*_max_, *K*_m_) under the quasi-steady-state assumption. While convenient, these simplifications hide the underlying microscopic steps – a measured *k*_cat_ is only an apparent aggregate of multiple steps rather than a true elementary rate. Such rate-law approximations can break down if their assumptions are violated (e.g., ignoring allosteric regulation or product inhibition). Using measured enzyme parameters in Michaelis–Menten form can reproduce full model dynamics well, but introducing too many coarse approximations degrades accuracy. Thus, finding the right balance between mechanistic detail and simplification is a persistent challenge.

Enzyme kinetic parameters are sensitive to temperature and pH, yet most models assume constant conditions. In reality, each enzyme has an Arrhenius-type temperature dependence and an activity profile across pH due to protonation effects. Capturing these dependencies requires additional parameters (e.g., activation energies, pH optima) and increases model complexity.^141^ Without explicit temperature/pH corrections, a model calibrated at one condition may perform poorly at another. Incorporating temperature and pH effects into kinetic equations (or adjusting parameters for different conditions) remains an open challenge, especially for large-scale models that would need consistent adjustment across hundreds of enzymes.

Bridging detailed enzymatic kinetics with system-wide or genome-scale models is extremely challenging. Genome-scale metabolic models can include thousands of reactions, far too many to parametrize individually with mechanistic kinetics given current data limitations. As a result, most of these models rely on steady-state assumptions without explicit kinetics. Efforts to build large-scale kinetic models must confront a parameter explosion and numerical stiffness. A true fully parametrized whole-cell kinetic model is still beyond reach in most cases.

### 3.8 Methodological Gaps

Beyond the intrinsic complexity of many chemical systems, methodological limitations in the underlying theory, automation, and uncertainty handling still constrain the scope and robustness of kinetic modeling.

Electronic structure methods remain a bottleneck in some cases. Accurate prediction of barrier heights and thermochemistry for systems with excited states, strong MR character, or large active spaces, such as in organic photochemistry, plasma chemistry, or heterogeneous catalytic interfaces, requires high-level methods like CASSCF, CASPT2, or coupled-cluster approaches. However, these become computationally intractable for larger systems, and more affordable scalable methods often sacrifice either accuracy or size-extensivity. MR PESs can be especially problematic for automated kinetics workflows, as there are few generally reliable protocols for balancing accuracy with tractability across diverse topologies.

Automated transition-state (TS) searches, conformer sampling, and reaction pathway enumeration are now mature for small, rigid gas-phase species. Still, they remain brittle for liquids, solids, and conformationally flexible molecules. Challenges include mapping reaction atoms in 3D, locating TSs when multiple solvent configurations are relevant or when microsolvation plays a significant role in the TS, dealing with extended periodic systems in surface catalysis, and sampling feasible partition functions for flexible species with strongly coupled large-amplitude internal rotations. Methods to generate statistically rigorous thermodynamic data for such species, especially where internal rot-vibrational coupling is strong, are still far from routine.

In addition to the poor scaling of single-point quantum chemistry calculations with the number of electrons, the computational demand grows very rapidly because the number of conformers depends exponentially on the number of large-amplitude coordinates. In addition, the number of possible reactions (and so also number of possible products) of large molecules also grows exponentially with molecular size. To keep the computational cost manageable, calculations on reactions of large molecules normally must be done using a combination of methods: barrier height estimates to narrow down the number of reactions to consider, force fields to identify the important conformers (and perhaps also to estimate partition functions), DFT calculations to compute the geometries and frequencies of stationary points, and high-level coupled-cluster or MR calculations for energies of the important stationary points. A failure in any of the methods can cause problems and, if the failure is not detected, the reported numbers from the computations may be misleading. For a recent example where failures in hundreds of published DFT calculations (due to high electron correlation) were not detected for 5 years, see ref 142. We suspect that the literature contains many other examples where one step in a multistep workflow failed but was not detected. As we move into an era of high-throughput calculations and large data sets, improved methods for error checking are needed, since it is often not practical to check for all possible errors in a large number of complicated calculations by hand. The “many-model”^108^ problem is another manifestation of the difficulty of ensuring that all the details of a large kinetic model are correct. Shared software and shared well-documented databases can make it easier for other groups to use and improve data sets and models developed by others, and would facilitate comparisons between kinetic models and a large number of experimental data sets.

While physics-based, rule/template mechanistic models remain indispensable, a purely postdictive mechanistic pipeline is increasingly unsustainable for prediction as the chemical space widens. With the combinatorial growth of the reaction space and insufficient parameter provenance, unknown channels and weakly constrained rates accumulate faster than rules and databases can be curated. Calibrated mechanisms thus become primarily explanatory: they rationalize existing observations or interpolate near their training regime, yet struggle to propose under-represented chemistry.^2^

While ML and active learning have been introduced to accelerate PES exploration and TS searches, these tools are not yet black-box reliable for nonspecialists, and they often require expert curation and retraining for new chemical spaces.

UQ remains underdeveloped in practice. Although local and global sensitivity methods, Bayesian calibration, and polynomial chaos expansions exist, their adoption is hampered by missing fundamental metadata in published mechanisms (e.g., parameter provenance, uncertainty ranges, and correlations). Without this information, rigorous propagation of uncertainties from the electronic-structure level through to reactor-scale predictions is rarely feasible.

Finally, there is cultural reporting bias in the field. Benchmark studies and successful predictions are overrepresented in the literature, while failed predictions and out-of-domain behavior are often omitted. This bias obscures the boundaries of current capabilities, limits community learning from failures, and slows the development of tools that explicitly handle uncertainty and quantify prediction reliability.

Overall, improving robustness in automated TS search, developing feasible partition function treatments for highly flexible molecules, in the gas-phase as well as in solution, and embedding traceable UQ into workflows are critical steps toward more predictive, transparent, and transferable kinetic models.

### 3.9 Summary

Despite major advances, significant gaps remain in our ability to model complex chemical systems predictively. Challenges span from fundamental theory, such as accurately treating MR electronic structures, to practical limitations in automation, UQ, and data availability. Real-world systems, whether involving condensed phases, heterogeneous surfaces, charged interfaces, plasmas, or enzymatic networks, introduce coupling between chemistry and physics that current methods only approximate. Addressing these limitations will require both methodological innovation and a broader adoption of transparent, uncertainty-aware modeling practices. Kinetic modeling has grown powerful, but accurately predicting complex, coupled, and out-of-equilibrium chemistry remains a frontier.

## 4 Where We Need to Go

The successes achieved in gas-phase combustion provide a foundation that the community increasingly recognizes as transferable to more complex and diverse domains. As capabilities in automation, robustness, and UQ continue to mature, similar approaches are being explored for heterogeneous catalysis, electrochemistry, liquid- and solid-phase chemistry, and even biological systems. Many envision automated, first-principles-based workflows that can extend predictive modeling across scales and disciplines, ultimately enabling decision-grade models with quantified uncertainties in areas far beyond traditional gas-phase combustion and pyrolysis.

As a community, we continue to push methods toward the dual goals of accuracy and scalability. Gas-phase kinetics has demonstrated the predictive power of high-level theory, yet extending this success to larger and more complex systems requires new strategies. While coupled-cluster and MR methods define the standard for small molecules, scalable treatments of excited states, condensed-phase reactions, and surface chemistry remain areas of active development. Exploring PESs remains a bottleneck, particularly for conformationally rich molecules, extensive networks, and multistate pathways such as photochemistry and plasma kinetics. Promising directions include enhanced sampling and metadynamics approaches coupled more tightly to statistical mechanics and transition-state theory, especially for liquids and interfaces where entropy and solvation effects dominate. Equally, we see the value of embedding UQ throughout, so that our predictions carry clearly quantified error bars and can be trusted as decision-grade tools. Automation has already reshaped our field, with gas-phase combustion leading in automated mechanism generation, accurate parameter computations, and model reduction. Extending automation into more complex chemistries motivates the development of tools for automatic conformer and transition-state searches in solvents and on surfaces, as well as ML surrogates that accelerate exploration and guide active learning.

Our successes to date highlight the importance of making predictive kinetics broadly accessible. Efforts such as RMG and Cantera have lowered barriers, but the community increasingly sees the need for a wider, more connected ecosystem spanning combustion, catalysis, electrochemistry, and biochemistry. To enable broader adoption, we are working toward tools that are easier to use, more extensively documented, and supported by sustainable open-source development. Graphical interfaces, APIs (application programming interfaces), and standardized data structures are helping experimentalists and engineers engage with predictive modeling directly. Reproducible workflows and shared benchmarks make it possible for us to improve collaboratively and to extend the reach of predictive chemical kinetics beyond specialists.

Predictive kinetics is increasingly a lever for industrial impact: better liquid-phase models can shorten pharmaceutical design–make–test cycles, improve process-transfer success, and reduce late-stage failures and overall cost of goods. Electrochemical models that capture potential-dependent kinetics and degradation guide electrolyte/electrode design, may positively impact battery lifetime, enable fast charging, and inform electrolyzer operation toward lower levelized H_2_ costs. Advances in catalysis and microkinetics enable lower temperature, electrified, and CO_2_-utilizing routes that reduce plant energy intensity and accelerate decarbonization across ammonia, methanol, and olefins. Across sectors, decision-grade models with quantified uncertainties serve as “virtual pilots” that derisk CAPEX and OPEX by shrinking piloting cycles and preempting scale-up surprises. Beyond design, they enable the real-time control and optimization of systems that remain only partially understood—bridging the gap between fundamental reaction understanding and robust plant operation under tight regulatory, supply chain, and sustainability constraints.

Theoretical and computational advances remain essential to move from empirical fitting to first-principles prediction. Priorities include scalable, size-extensive levels of theory for excited and condensed-phase species; faster, higher-fidelity PES exploration for multiconformer and multistate chemistry; and tighter integration of enhanced-sampling methods with statistical mechanics and TST, particularly for liquids and interfaces where entropy and solvation dominate.

Data-driven and physics-constrained ML interatomic potentials are accelerating PES evaluation by orders of magnitude while retaining near ab initio fidelity. These models enable long-time, large-system reactive molecular dynamics that can uncover pathways (e.g., rare events, surface-mediated sequences, solvent-assisted mechanisms) that are expensive to sample with on-the-fly electronic structure. Hybrid strategies that learn corrections to mechanistic models (e.g., Δ-learning for barriers, solvation and field effects, or coverage-dependent lateral interactions) would provide the best of both worlds, provided their uncertainties are tracked and propagated. Establishing community benchmarks and stress tests that compare these paradigms across chemistries and conditions will be crucial for trustworthy deployment. Such benchmarks must incorporate robust data governance practices to prevent train/validation leakage and enable out-of-domain applicability detection. For long-term impact, the community will also need to establish standards for versioning and ensuring the archival reproducibility of these learned models.

Automation has transformed model construction, but static pipelines alone are insufficient for some of the field’s important challenges. A practical way forward could be pairing today’s automated pipelines with a reasoning agentic layer, as detailed elsewhere.^143^ In this view, model development runs on two tightly coupled lanes: a fast execution lane that automates what we already do well (mechanism growth, parameter refinement, reduction, and simulation) and a deliberative agentic lane that plans computations and experiments, chooses among tools, revises hypotheses, and allocates budget based on uncertainty and expected information gain.^143^ Agentic systems should be coupled to self-driving laboratories and partner facilities to run closed-loop cycles in which models propose experiments, fundamental experiments update models, and policy stop-rules enforce safety and budget constraints. If we adopt these practices, predictive chemical kinetics can shift from automation to an autonomous enterprise. This envisioned transparent, adaptive, and community-auditable autonomous approach could free researchers to focus on insight rather than workflow management.

We see a chance to make our collective effort even more effective. Some of the hardest work (high-quality data curation, cross-code round-robins, shared benchmarks, and replayable provenance) remains essential yet often under-recognized. By visibly crediting these contributions and organizing friendly community challenges where theory and experiment meet under common protocols, we can turn today’s bottlenecks into shared assets. The aim is to broaden scopes of future studies toward rigorous and decision-grade models that accelerate science.

As applications broaden, so do the systems we aim to model. In electrochemistry, predictive kinetics depends on capturing potential-dependent energetics and electron-transfer processes; in biochemistry and pharmaceuticals, modeling medium-sized organic molecules in complex solvents could reshape synthesis planning and drug discovery. Environmental and geosciences pose additional challenges, from subsurface H_2_ generation to CO_2_ mineralization over geological time scales. At the same time, we are exploring new synergies between automated modeling, laboratory robotics, and closed-loop experimentation, creating feedback cycles where models inform experiments and experiments refine models.

Technical advances are most impactful when paired with evolving practices within the community. Increasingly, we recognize the value of publishing not only successes, but also failures, reporting uncertainties alongside central predictions, and emphasizing forward-looking applications rather than purely retrospective fits. Open-source platforms should continue to receive sustained support, have clear documentation along with examples spanning diverse systems. Shared benchmark sets across chemistries and conditions, combined with FAIR (findability, accessibility, interoperability, and reusability) data practices and standardized thermokinetic schemas, are helping us compare methods systematically and reuse results effectively. Tools must support standardized data structures, versioning, and reproducibility, facilitating collaborative model development and benchmarking. By adopting these practices collectively, we strengthen the foundation for moving from an explanation of past observations to a reliable prediction of future behavior.

## 5 Perspective

Chemical kinetic modeling is at an inflection point. The past two decades have delivered robust workflows – from automated ab initio partition functions and molecular properties to TST/ME rate theory, automated mechanism construction, and multiscale validation – that make quantitative prediction routine in well-characterized regimes. At the same time, the questions we aim to answer now span chemistries, phases, and length–time scales that stretch those workflows beyond their comfort zone. Meeting that opportunity will depend as much on how we practice modeling as on the next methodological breakthrough.

A first step is being clearer about where our models do and do not generalize. Many of us can point to cases where models agree closely with data, and to others where they do not. However, the latter are underreported and under-curated. Sharing negative results, parameter provenance, and uncertainty ranges alongside successful benchmarks helps set realistic expectations and accelerates progress. Clearly quantified error bars, uncertainties traced back to data and level-of-theory choices, allow models to be used as decision tools rather than just explanatory ones.

A second step is shifting emphasis from retrospective explanation to prospective prediction. When we test models on conditions and chemistries not used in their construction, we learn which assumptions travel well, and which do not. Community benchmark suites that span gas, liquid, surface, electrochemical, and plasma environments, paired with reproducible workflows, are needed.

Lowering barriers remains essential. The ecosystem around RMG, AutoMech, Cantera, T3 and related tools demonstrates how open, well-documented software broadens participation. Extending that usability across domains (electrochemistry, solvated and interfacial chemistry, biochemistry) with stable APIs, clear examples, and FAIR data standards will help experimentalists and process engineers use predictive kinetics directly, while preserving scientific rigor.

Finally, automation has evolved from scripted pipelines to more adaptive systems. Static automation already links mechanism generation, sensitivity-driven ab initio refinement, pressure-dependence rate coefficient refinement, and validation. Looking ahead, agentic workflows that can reason about goals, choose tools, track provenance, and actively design informative experiments offer a path to faster, more reliable convergence, especially in complex, multiphysics settings. Pairing these agents with explicit uncertainty handling and shared benchmarks can turn today’s best practices into scalable, cross-domain capability.

In short, we have the ingredients – sound theory, growing automation, open tools, and rich data – to extend predictive kinetics well beyond its traditional strongholds. By emphasizing reproducibility, uncertainty transparency, successes and failures alike, accessible software, and forward-looking validation, we can carry that strength into the more complex chemistries that matter next.
